# Grape seed extracts inhibit dentin matrix degradation by MMP-3

**DOI:** 10.3389/fphys.2014.00425

**Published:** 2014-10-31

**Authors:** Mayssam Khaddam, Benjamin Salmon, Dominique Le Denmat, Leo Tjaderhane, Suzanne Menashi, Catherine Chaussain, Gaël Y. Rochefort, Tchilalo Boukpessi

**Affiliations:** ^1^EA 2496, Orofacial Pathologies, Imaging and Biotherapies, Dental school, University Paris Descartes, Sorbonne Paris CitéMontrouge, France; ^2^Assistance Publique - Hôpitaux de Paris, Odontology Departments (Bretonneau and Charles Foix)Paris, France; ^3^Medical Research Center Oulu, Institute of Dentistry, Oulu University Hospital and University of OuluOulu, Finland; ^4^Laboratoire CRRET, Université Paris-Est, Centre National de la Recherche ScientifiqueCréteil, France

**Keywords:** grape seed extracts, dentin, matrix metalloproteinases, caries, mouthrinse

## Abstract

Since Matrix metalloproteinases (MMPs) have been suggested to contribute to dentin caries progression, the hypothesis that MMP inhibition would affect the progression of dentin caries is clinically relevant. Grape seed extracts (GSE) have been previously reported to be natural inhibitors of MMPs.

**Objective:** To evaluate the capacity of a GSE mouthrinse to prevent the degradation of demineralized dentin matrix by MMP-3 (stromelysin-1).

**Materials and Methods:** Standardized blocks of dentin obtained from sound permanent teeth extracted for orthodontic reasons were demineralized with Ethylenediaminetetraacetic acid (EDTA) and pretreated either with (A) GSE (0.2% w/v), (B) amine fluoride (AmF) (20% w/v), (C) a mouthrinse which contains both, (D) placebo, (E) sodium fluoride (0.15 mg.ml^−1^), (F) PBS, (G) Chlorhexidine digluconate (CHX), or (H) zinc chloride (ZnCl_2_). The dentin blocks were then incubated with activated recombinant MMP-3. The supernatants were analyzed by Western Blot for several dentin matrix proteins known to be MMP-3 substrate. In parallel, scanning electron microscopy (SEM) was performed on resin replica of the dentin blocks.

**Results:** Western blot analysis of the supernatants revealed that MMP-3 released from the dentin matrix small proteoglycans (decorin and biglycan) and dentin sialoprotein (DSP) in the AmF, sodium fluoride, PBS and placebo pretreated groups, but not in the GSE and mouthrinse pretreated groups. SEM examination of resin replica showed that the mouthrinse and its active components not only had an anti-MMP action but also modified the dentin surface accessibility.

**Conclusion:** This study shows that GSE either alone or combined with AmF as in the evaluated mouthrinse limits dentin matrix degradation. This association may be promising to prevent the progression of caries within dentin. However, the procedure should be adapted to clinically relevant durations.

## Introduction

Matrix metalloproteinases (MMPs), also designated as matrixins, hydrolyze components of the extracellular matrix (Brinckerhoff and Matrisian, 2002) and play a central role in many biological and pathological processes. Their role in the dentin caries process has also been proposed in several studies (Tjaderhane et al., 1998; Sulkala et al., 2001). MMPs were shown to be expressed during tooth development and to be necessary for normal dentin formation (Bourd-Boittin et al., 2005). After dentin mineralization, they remain trapped in the calcified matrix either under active or proenzyme forms, which may explain their persistent presence within the dentin of adult teeth (Tjaderhane et al., 1998; Mazzoni et al., 2007). It can be therefore hypothesized that their exposure and activation during the caries process would allow these enzymes to promote matrix degradation and caries progression. Indeed, cariogenic bacteria create acidic environment, exposing the dentin matrix. However, cariogenic bacteria cannot degrade dentin matrix after demineralization (Katz et al., 1987), and the bacteria collected from dentinal lesions created *in situ* were unable to degrade collagen *in vitro* (van Strijp et al., 1997). Moreover, purified bacterial collagenases have low degrading activity in acidic environment (Clarkson et al., 1987; Kawasaki and Featherstone, 1997). It can be therefore assumed that the cariogenic demineralization process initiated by the bacteria re-exposes endogenous MMPs and also potentially induces their activation through the acidity they create (Tjaderhane et al., 1998; Sulkala et al., 2001), hence enabling further dentin matrix degradation. In addition, saliva, which bathes the dentin carious lesion, contains several MMPs that can also participate in the organic matrix degradation (Tjaderhane et al., 1998; van Strijp et al., 2003; Nascimento et al., 2011).

Intra-oral administration of modified tetracylines and zoledronate, known as chemical MMP inhibitors, delayed caries progression in rats (Tjaderhane et al., 1999; Sulkala et al., 2001). These studies suggest that MMPs have a deleterious effect in dentin caries and that MMP inhibition may represent a new avenue for the prevention or treatment of caries. Along this line, several synthetic MMP inhibitors are already in usage in the dental practice. They mainly include Ethylenediaminetetraacetic acid (EDTA) (Thompson et al., 2012), Chlorhexidine digluconate (CHX) (Gendron et al., 1999; Carrilho et al., 2007), or Zinc chloride (ZnCl_2_) (Toledano et al., 2012) whose MMP inhibitory effect is based on their zinc and calcium chelator properties (Chaussain et al., 2013). In addition, several natural molecules have been previously reported to have MMP inhibitory properties. Grape-seed extracts (GSE) were shown to suppress lipopolysaccharide-induced MMP-1 and MMP-9 secretion by macrophages in culture and to inhibit their activity, and were therefore recommended for use in the development of new treatments of MMP-mediated disorders such as periodontitis (La et al., 2009). Hence, the MMP-inhibitory effects of these natural substances and the fact that they are expected to cause only minimal side effects make them particularly attractive for the treatment of dentin caries, since they can be integrated into the daily-used topical products, such as toothpastes and mouthrinses, or into products used for direct application, such as varnish. In addition, increasing concentrations of GSE have been shown to decrease the degradation rate of the dentin matrix after bacterial collagenase treatment, indicating an inverse relationship between concentration of GSE and collagen solubilization (Bedran-Russo et al., 2008, 2011). Noteworthy, GSE induced collagen inter-microfibrilar cross-links at high concentration (6.5% w/v), modifying the tensile strength properties of the dentin matrix (Bedran-Russo et al., 2011).

Among the MMPs detected in dentin, Stromelysin-1, also termed MMP-3 or proteoglycanase, is capable of degrading the protein core of proteoglycans (PGs), resulting in the release of soluble glycosaaminoglycans (GAGs) (Hall et al., 1999). Boukpessi et al. have previously developed a simplified but well-controlled *in vitro* model to study the dentin caries process, consisting of the pretreatment of human sound dentin cubes by acidic or EDTA solutions followed by a MMP (MMP-3, MMP-2, or MMP-9) treatment of the demineralized matrix (Boukpessi et al., 2008). Using this model, they have shown that MMP-3 released from the collagen scaffold several non-collagenous proteins (NCPs) such as two Small Leucine-Rich Proteoglycans (SLRPs) decorin (DCN), and biglycan (BGN) as well as four members of the SIBLING (Small Integrin-Binding LIgant N-Linked Glycoproteins) family: dentin sialoprotein (DSP), matrix extracellular phosphoglycoprotein (MEPE), bone sialoprotein (BSP), and osteopontin (OPN) (Boukpessi et al., 2008). All these NCPs are known to be associated to the collagen fibers in the dentin (Orsini et al., 2007, 2008) and to participate in the initiation or control of dentin mineralization (Orsini et al., 2012).

Recently, a new daily mouthrinse composed of GSE and amine fluoride (AmF) has been developed. This combination showed both a significant antiplaque activity and an important antioxidant capacity *in vitro*, without any bactericidal effects (Furiga et al., 2014). The objective of this study was to evaluate the capacity of this mouthrinse to prevent dentin matrix degradation by MMPs. As GSE are known to be natural inhibitors of MMPs, we hypothesized that the tested mouthrinse has the ability to limit dentin matrix degradation following MMP treatment, mainly by reducing NCP release. In this study, MMP-3 was selected because we previously showed that it was the only MMP among those tested able to release several NCPs from the dentin matrix (Boukpessi et al., 2008).

## Materials and methods

### Samples selection

Sound permanent premolars extracted for orthodontic reasons were collected, gently cleaned with tap water and kept at −20°C. All teeth were collected with informed and oral consent from the patients and the parents according to ethical guidelines set by the French law (Loi Bioéthique n°2004-800) and with a special authorization for our team (n°DC-2009-927, Cellule Bioéthique DGRI/A5, Paris, France).

### Dentin demineralization

Enamel was removed by drilling with a high-speed diamond bur under water cooling. Standardized dentin blocks (1 mm^3^, 1.2 mg) were prepared from the outer part of teeth with a diamond disk saw under continuous water spray (Accutom 5, Streuers, Copenhagen, Denmark). Blocks were immersed individually at 4°C in 170 μl of 4.13% w/v EDTA (disodic salt) pH 7.2 for 7 days. The demineralizing solution was supplemented with 1/100 Proteinase Inhibitor Cocktail Set V EDTA free (Calbiochem, La Jolla, CA). After demineralization, EDTA was removed by rinsing the blocks three times in PBS.

### Pretreatment of dentin blocks with different solutions

Dentin blocks were incubated 48 h at 4°C in different solutions according to the treatment group. The mouthrinse EludrilDAILY® (Pierre Fabre Santé, Castres, France), containing 2 mg.ml^−1^ of GSE (0.2% w/v, Vitimed, Vallon Pont d'Arc, France) and 10.2 mg.ml^−1^ of Fluorinol®, was used as pretreatment solution for group A. The active principles of the mouthrinse were tested separately at the same final concentration found in the mouthrinse: GSE solution (2 mg.ml^−1^, Pierre Fabre Santé) for group B, and AmF (Fluorinol®, 20% w/v; Pierre Fabre Santé) for group C. Placebo, which has the same composition that the mouthrinse without the active principles (GSE and Fluorinol,) was used for group D, sodium fluoride (NaF, N°B48-1450-038 0.15 mg.ml^−1^) for group E, PBS for group F, dissolvent composition of 5% CHX (w/v, pH 6, Pierre Fabre Santé) for group G, and ZnCl_2_(Sigma-Aldrich) with a final concentration of 3.3 mg.ml^−1^ pH 6.5, for group H.

### Action of MMP-3 on pretreated dentin

Recombinant human MMP-3 (R&D Systems Inc., Minneapolis, MN, USA) was first incubated with aminophenylmercuric acetate (1 mM, 1 h, 37°C) to achieve activation. Each demineralized and pretreated dentin block was incubated with the activated MMP-3 (1 μg/ml) in a 120 μl buffer containing 100 mM Tris pH 7.2; 0.15 M NaCl; CaCl_2_ 5 mM; 0.05% w/v Brij 35, and 0.02% w/v NaN_3_ (SIGMA, St Louis, MO, USA). Following 72 h incubation at 37°C, the supernatants were subjected to Western blot analysis to reveal the proteins released by MMP-3 treatment.

### Western blot analysis

Laemmli-buffer-diluted samples were electrophored on 4–20% w/v gradient gels (Mini-Protean® TGX™ gels, BioRad, Marne la Coquette, France) and separated proteins were transferred to a nitrocellulose membrane (Trans-Blot Transfer Medium, BioRad). The membrane was blocked with 5% v/v bovine serum and incubated overnight at 4°C with one of the following polyclonal antibodies: anti-DCN (LF 113), anti-BGN (LF 159) (kind gifts from Larry Fisher, NIH, Bethesda, MD, USA) used at 1 mg.ml^−1^ and diluted at 1/1500; anti-DSP (LF 153) and anti-MEPE (kind gift from Peter Rowe, Kidney Institute, Kansas City, KS, USA) were used at 1 mg.ml^−1^ and diluted at 1/700. After incubation with a peroxidase-conjugated swine anti-rabbit IgG at 1/10000 dilution for 1 h at room temperature, the membrane was developed by means of the Renaissance Western Blot Chemiluminescence Reagent Plus Kit (NEN Life Science, Boston, MA, USA). Chemiluminescent signals were digitalized and the relative intensity of the different bands was compared using ImageJ software (v1.45p, NIH).

### Statistical analysis

For each experiment, samples were performed in triplicate for each condition and repeated three times. The values found in each experiment were averaged, and the mean was used for comparison between groups. Intergroup comparison was performed by one-way analysis of variance with Bonferroni correction (α = 0.05). If a statistical difference was detected, a *post hoc* non-pairwise multiple comparison (Scheffe's test) was used to identify differences among the groups. Data are expressed as mean ± SD.

### Scanning electron microscopy analysis (SEM)

To investigate whether the different tested pretreatments have influence on demineralized dentin treated by MMP-3, SEM analysis was performed on the replicas of the dentin blocks. Dentin block surfaces were treated with phosphoric acid 40% (w/v, Onyx ™ Centrix, Shelton, CT, USA) for 20 s. After rinsing and gentle drying, a thick layer of dental adhesive resin (OptiBond® SoloPlus Kerr Corporation, Orange, CA, USA) was placed on dentin. After photopolymerization (Free light 3M ESPE, Seefeld, Germany), a layer of microhybrid flowable composite (Tetric EvoFlow®, Ivoclar Vivadent, Saint Jorioz, France) was placed and photopolymerized. The dentin block was dissolved with HCl 8N during 36 h. Replica surfaces were rinsed with flow water, air-dried, glued with carbon adhesive discs on aluminum stubs, sputter-coated (SC5OO, BIO-RAD Laboratories, Hercules, CA, USA) with palladium/gold (20 mA, 0.1 Tor, 2 min). The replicas were observed by SEM (EVO MA 10 SEM, Carl Zeiss, Oberkochen, Germany) equipped with tungsten gun and Everhart-Tornley secondary electron detector.

## Results

### Cleavage of dentin matrix components by MMP-3

**Figure 1:**
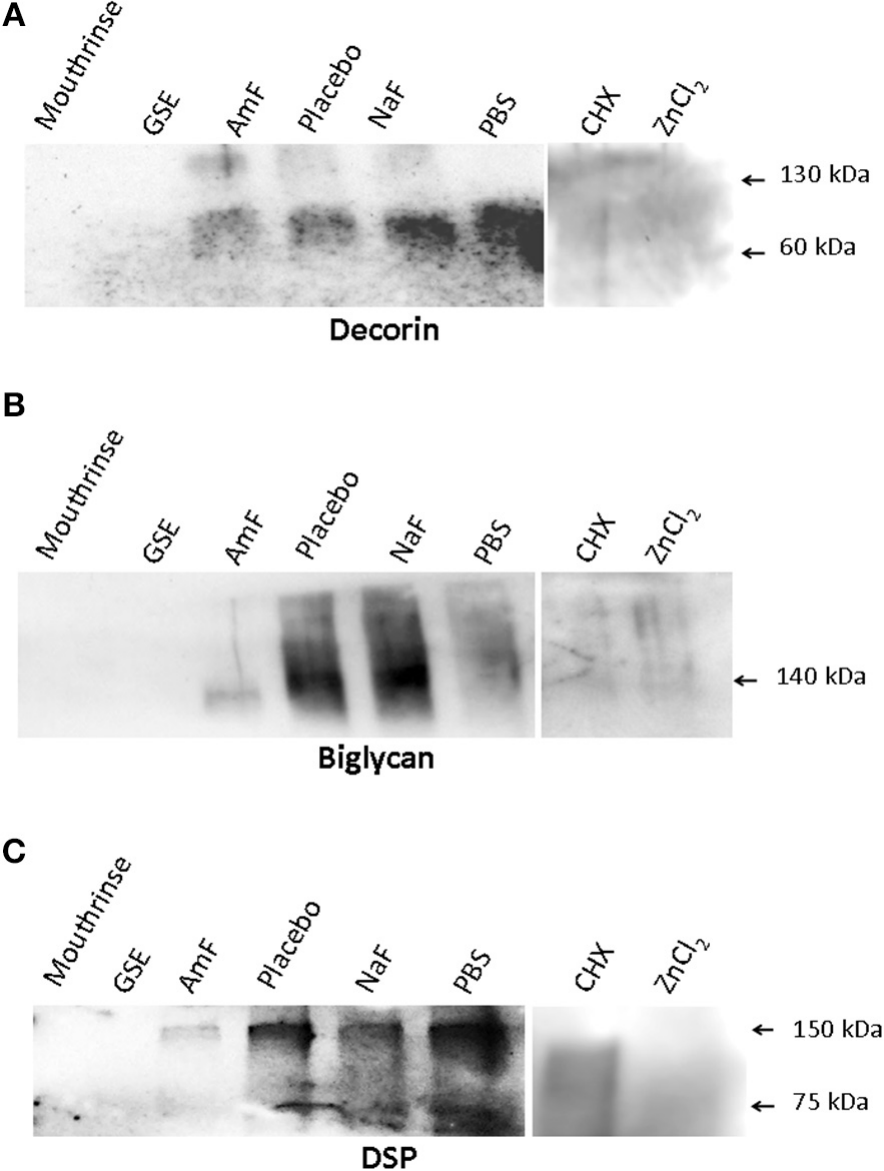
**Western blot analysis assessing the ability of the tested Mouthrinse to prevent NCP release. (A)** DCN is detected as a main band at 60 kDa in the following groups: pre-treatment with amine fluoride (AmF), placebo, NaF, and PBS. An additional fainter band is seen at 130 kDa in the AmF and placebo groups. No band is observed in the Mouthrinse, GSE, CHX, and ZnCl_2_ pre-treated groups. **(B)** BGN is detected as a large band around 140 kDa in placebo, NaF, and PBS pre-treated groups. This band appears fainter in the AmF group. No band is observed in the Mouthrinse, GSE, CHX, and ZnCl_2_ pre-treated groups. **(C)** Dentin sialoprotein (DSP) is observed at 150 and 75 kDa in the AmF, placebo, NaF and PBS pre-treatment groups. A smeary signal is detected in the CHX pre-treated group whereas no band is present in the Mouthrinse, GSE, and ZnCl_2_ groups.

**Figure 2:**
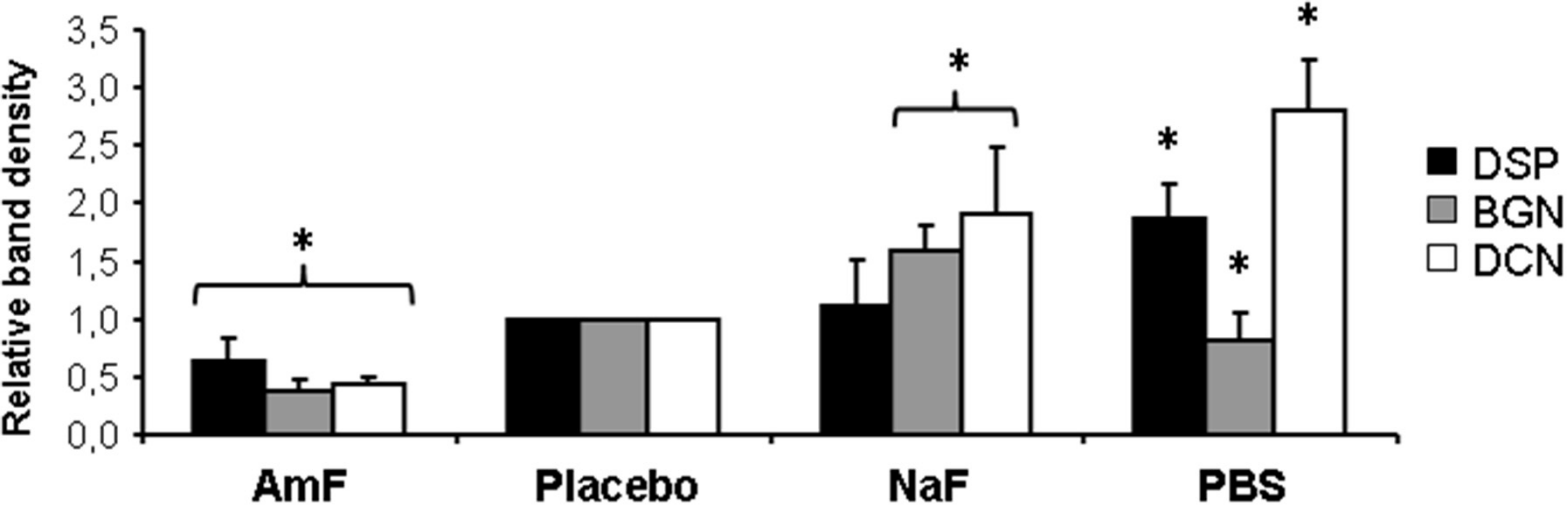
**Quantification of NCP release from Western blot analysis**. Quantification of the different bands by ImageJ after normalization with placebo bands shows a significant weaker protein release in samples pre-treated with AmF (all proteins tested) and PBS (BGN), and a significant stronger release in NaF (BGN and DCN) and PBS (DSP and DCN) pre-treated groups. Black bars: DSP; gray bars: BGN; white bars: DCN. ^*^p < 0.05 vs. placebo value.

Western blot analysis of supernatants retrieved from the samples pre-treated with mouthrinse and GSE, followed by MMP-3 treatment, did not reveal any detectable DCN band (Figure 1). In contrast, DCN was visualized as a large band around 60 kDa in the AmF, placebo, NaF, and PBS pre-treated groups, and as an additional weaker band at 130 kDa mainly detectable in the AmF and placebo pre-treated group (Figure 1A). BGN was detected as a single large band around 140 kDa in the supernatants of the samples pre-treated with AmF, NaF, placebo, and PBS, but not with the mouthrinse and GSE (Figure 1B). For both SLRPs, no band was detected in the CHX and ZnCl_2_ pre-treated groups (Figures 1A,B). DSP was also released by MMP-3 and was observed as two bands around 150 and 75 kDa in the AmF, placebo, NaF and PBS pre-treated groups. No signal was detected in the mouthrinse, GSE and ZnCl_2_ supernatants, whereas a smear labeling was seen with CHX pre-treatment (Figure 1C). Quantification of the different bands after normalization with placebo is showed in Figure 2. In particular, release of DSP after pre-treatment with AmF was around 0.6 (*p* < 0.05), compared to release of DSP in placebo (set at 1), and release of BGN and DCN after pre-treatment with AmF were both around 0.4 (*p* < 0.05). Contrary to this, release of BGN and DCN after NaF pre-treatment were significantly higher compared to placebo (1.6 and 1.8, respectively, *p* < 0.05). At last, release of BSP and DCN after pre-treatment with PBS were significantly higher compared to placebo (1.8 and 2.8, respectively, *p* < 0.05) whereas release of BGN was significantly lower compared to placebo (0.9, *p* < 0.05). For MEPE, no signal was detected (data not shown).

### Effect of pretreatments on dentin penetration by low viscosity resin after MMP-3 treatment

**Figure 3:**
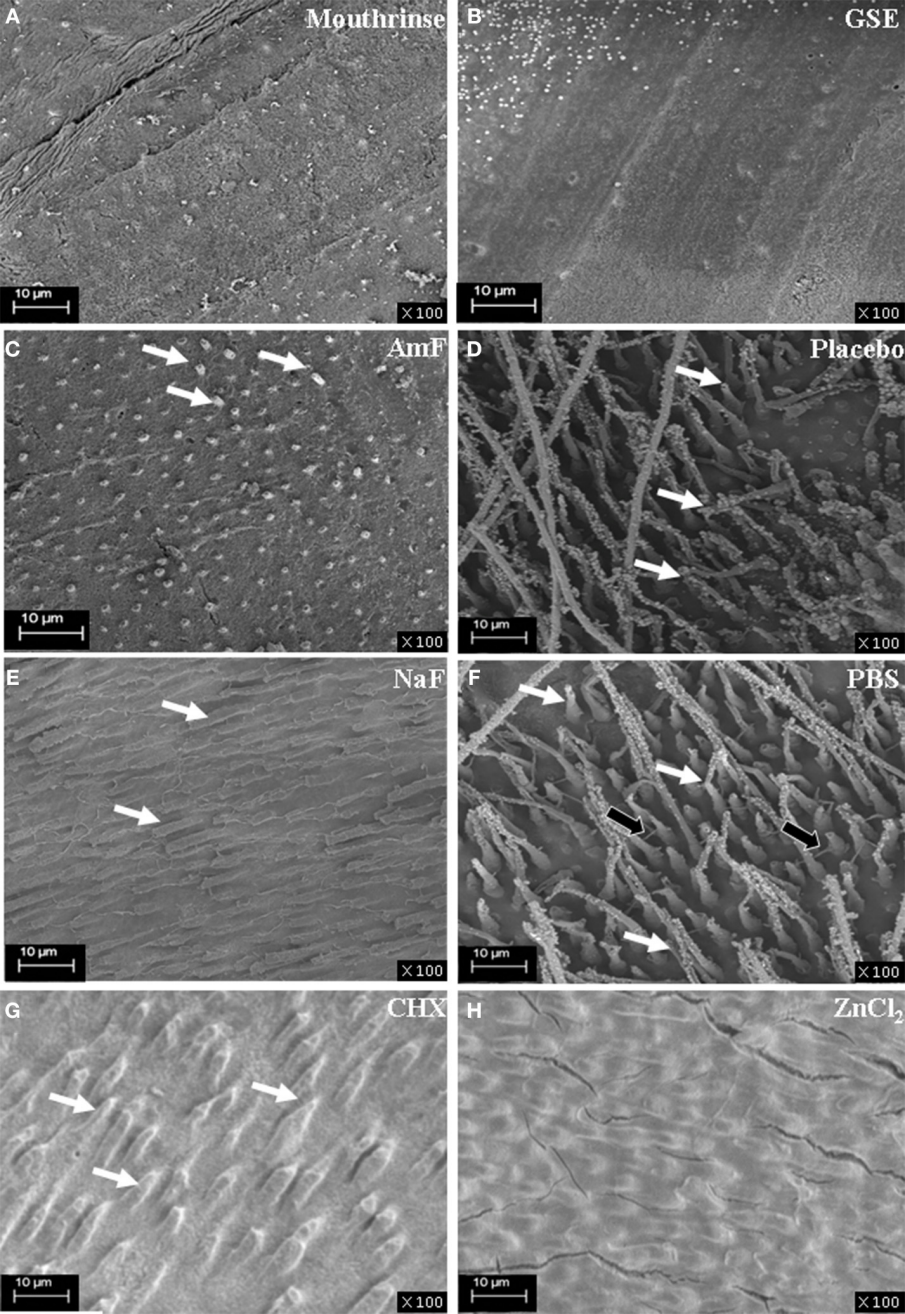
**SEM analysis of resin replicas of the demineralized, pre-treated and MMP-3 treated dentin blocks**. The resin penetration is absent in the mouthrinse groups **(A)** and GSE **(B)** as no tubule is enlarged by the MMP-3 treatment and penetrated by resin; a regular dentin surface is observed. In the AmF group **(C)**, sparse penetrations of resin are seen (white arrows). In the NaF group **(E)**, mildly infiltrated dentinal tubules are visualized (white arrows). In contrast, a very deep penetration of the resin within the tubules (white arrows) is observed in the placebo **(D)**. In addition to a deep penetration of the tags within the tubules (white arrows) in the PBS group **(F)**, the filling of lateral secondary dentin tubules is observed (black arrows). A faint disorganization of the dentin architecture is seen in CHX pretreated samples (white arrows) **(G)**. ZnCl_2_ pretreated replicas show a regular and smooth dentin surface **(H)**.

Boukpessi et al. have previously shown that MMP-3 treatment, by removing some of the matrix components, improved the diffusion of low-viscosity resin into the dentin sub-surface (Boukpessi et al., 2008). Resin penetration into the dentin blocks was analyzed after MMP-3 treatment in the different groups (Figure 3). In the mouthrinse (Figure 3A) and GSE (Figure 3B) pretreatment groups, the tubules were not enlarged by the MMP-3 treatment and were not penetrated by the resin. Limited resin penetrations were observed in both fluoride pretreated groups (Figures 3C,E), but they were more pronounced in the NaF group. In contrast, a very deep penetration of the resin tags within the tubules was observed in the placebo and PBS groups (Figures 3D,F). With the two MMP inhibitors pretreatment, a faint disorganization of the dentin architecture was observed in CHX pretreated group (Figure 3G), whereas ZnCl_2_ pretreated replicas showed a regular dentin surface (Figure 3H). Hence, the mouthrinse and its active components appear to limit the disorganization inside dentinal tubules induced by the MMP-3 action.

## Discussion

**Figure 4:**
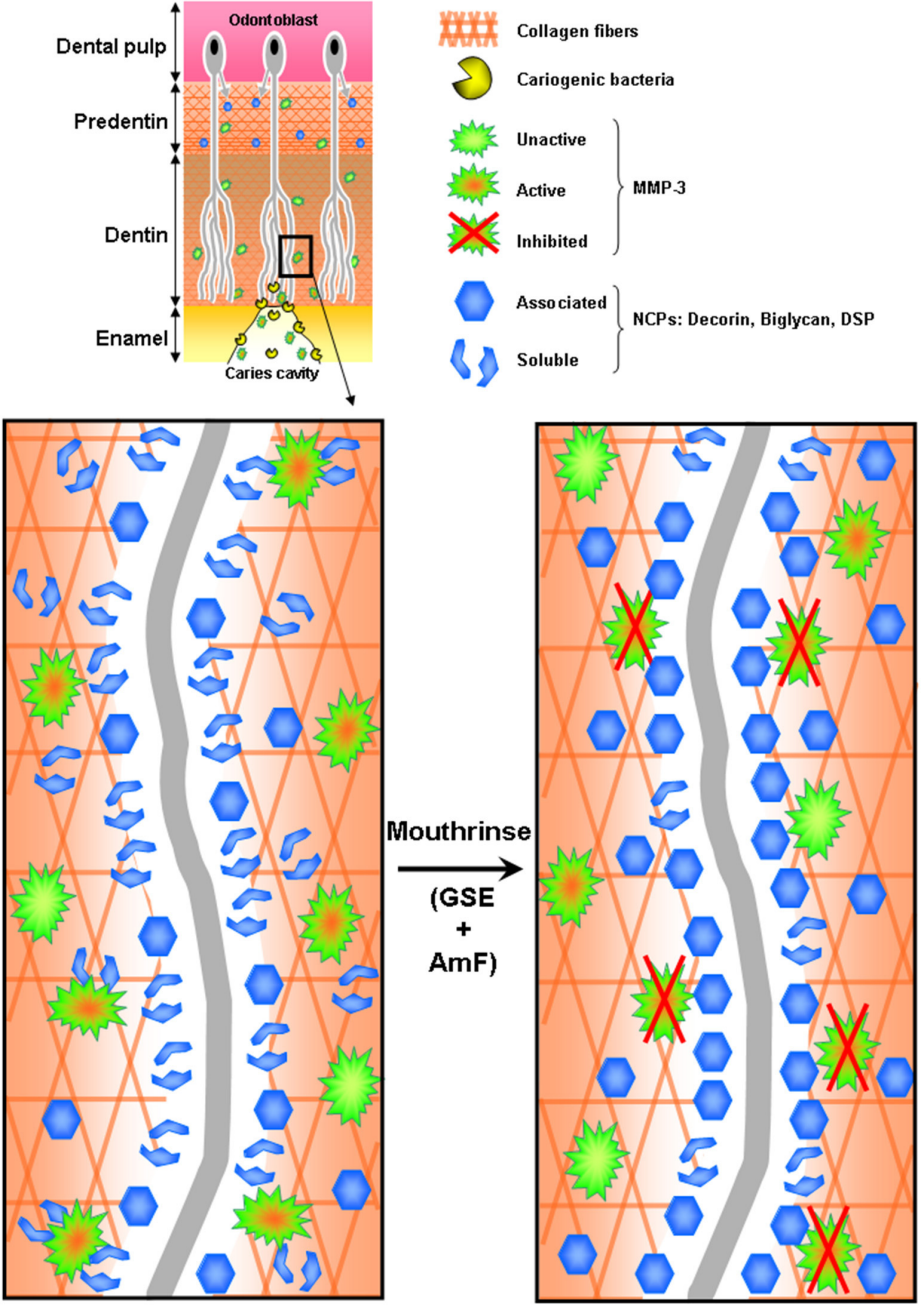
**Hypothetic representation of MMP-3 inhibition by the tested mouthrinse during the dentin carious process**. Cariogenic bacteria present in the caries cavity release acids that demineralize the dentin matrix and activate host dentin or saliva-derived MMPs, including MMP-3. Activated MMP-3 then releases from the collagen scaffold several non-collagenous proteins (NCPs): DCN, BGN and some Sibling proteins. Mouthrinse, composed of GSE (Grape Seed Extracts) and AmF is an efficient MMP inhibitor that prevents the release of NCPs associated to the collagen fibers in the dentin.

Our results show that dentin pretreatment with the tested mouthrinse, and to some extent with its active principles, limits dentin matrix disorganization induced by MMP-3, especially along the dentinal tubules (Figure 4). In this study, we used an *in vitro* model combining a demineralization process and a subsequent MMP-3 treatment in order to test MMP inhibitors on dentin matrix degradation (Boukpessi et al., 2008). MMP-3 has been identified in predentin (Hall et al., 1999) and in dentin (Boukpessi et al., 2008), where it localizes within the intertubular dentin, along the collagen fibrils (Mazzoni et al., 2009). *In vitro* human demineralized dentin has been extensively used as a model to study the caries process (Ten Cate et al., 1991). However, demineralized dentin represents a simplified model of the caries process (Ten Cate, 2001), as it does not take into account the degradation of the organic matrix by endogenous proteinases and/or by bacterial collagenases. Therefore, the selected model would be more relevant to mimic dentin matrix degradation and to test the effect of MMP inhibitors.

The reported roles for DCN and BGN include collagen stabilization, collagen fibrillogenesis, calcium binding potential, interaction with hydroxyapatite, as well as crystal growth inhibition. DCN is strongly expressed over the mineralized dentin (mainly inside the dentinal tubules) and in the predentin adjacent to the mineralization front, whereas BGN is mainly expressed in predentin during matrix formation and maturation (Mazzoni et al., 2012; Orsini et al., 2012). DSP, abundantly expressed in dentin and not detected in predentin, may be essential in the biomineralization process during the dentin formation (Mazzoni et al., 2012; Orsini et al., 2012). The prevention of these NCPs release may protect further matrix degradation by exposing the collagen fibers to more collagen-specific MMPs such as collagenases and gelatinases (Malla et al., 2008) which are also present in the dentin organic matrix and in the saliva (Tjaderhane et al., 1998).

Our Western blot results showed that the pretreatment of the demineralized dentin with either GSE or the mouthrinse inhibited the MMP-3-dependent release of two SLRPs and the SIBLING member DSP, a specific dentin NCP. It should be noted that the molecular weights of these glycoproteins released by MMP-3 are somewhat different from those of their purified forms. This may be due to their release either as dimers or as molecular forms still bound to fragments of other matrix components to which they are bound in the dentin. In fact, DCN, which is reported in the literature at 45 kDa (Embery and Rolla, 1980), appeared on western blot at 60 kDa suggesting a protein complex. BGN, whose monomeric form is reported at 45 kDa (Embery et al., 2001), was detected as a band at 140 kDa. Interestingly, studies on the folding and stability of DCN and BGN implicated their organization as complex both in solution and as crystal structure, which is consistent with our result (Scott et al., 2006). For DSP, the molecular weights observed in the present study, 150 and 75 kDa, are higher than those reported in the literature (95 and 53 kDa) (Ritchie et al., 1995). It can be proposed that MMP-3 releases these proteins by degrading their attachment to the dentin extracellular matrix.

The release of NCPs visualized by Western blot is in accordance with SEM exploration of the dentin surfaces showing that the pretreatment with the mouthrinse and GSE both inhibited the enlargement of the dentinal tubules by the enzymatic action of MMP-3. This approach may be beneficial in terms of initial dentin caries prevention, but could also improve life expectancy of adhesive-resin interfaces. Indeed, previous studies have indicated that the use of MMP inhibitors prevent early degradation of resin-dentin bonds by endogenous MMPs (Sabatini and Patel, 2013) suggesting therefore that GSE could be used to treat the dentin surface of the cavity before the adhesive procedures. However, the consequences of this treatment on resin adhesion to the dentin surface still remain to be assessed by additional mechanical testing complemented by failure analysis.

GSE has been suggested to have an additional inhibitory effect on the demineralization process. It has been shown to inhibit the demineralization and/or to promote the remineralization of artificial root carious lesions *in vitro* under dynamic pH-cycling conditions (Xie et al., 2008). In addition, GSE treatment was also shown to increase the microhardness of the lesions in these conditions. Xie et al. suggested that GSE could contribute not only to the deposition of mineral on the superficial layer of the lesion, but may also interact with the organic portion of the root dentin through Proanthocyanidin-collagen interaction, stabilizing the exposed collagen matrix (Xie et al., 2008). Noteworthy, during the production of replicas, the 40% phosphoric acid dentin treatment opened the dentinal tubules only in placebo and PBS groups. This finding suggests that the mouthrinse and its active principles' pretreatment not only had an anti-MMP action but also modified the dentin surface accessibility (Bachelar-Sá et al., 2014). Along this line, dentin treatment with GSE has been reported to limit the matrix degradation by specific bacterial proteases through induction of collagen inter-microfibrilar cross-links by removing and replacing GAG by GSE (Bedran-Russo et al., 2008, 2011). At the concentration used in the present study (0.2%), it can be hypothesized that both an anti-MMP effect and a surface modification are achieved to protect the dentin matrix.

Regarding the effect of the other MMP inhibitors used, our results show that ZnCl_2_ pretreatment was also effective in protecting dentin from cleavage by MMP-3 as there was no detectable release of matrix components, while with CHX pretreatment there was only a slight release of DSP. Interestingly, Toledano et al. have previously reported that these two MMP inhibitors prevented collagen degradation by endogenous MMPs in dentin beams pretreated with acidic solutions and incubated in artificial saliva (Toledano et al., 2012). However, only the ZnCl_2_ pretreatment maintained a total inhibitory effect after a prolonged incubation period in the artificial saliva. Both approaches, conducted in different models, suggest that ZnCl_2_ may be a more efficient MMP inhibitor over time than CHX. AmF, one of the active principles composing the tested mouthrinse, was a better inhibitor of MMP-3 in the present model than NaF as it decreased the release of DCN, BGN, and DSP from the matrix and prevented dentinal tubules enlargement. These protective effects on the dentin matrix were not observed with NaF pretreatment. Accordingly, Mei et al. compared MMP-inhibitory effects of several organic and inorganic fluorides by gelatin zymography and showed that the silver diamine fluoride, an organic fluoride, was the most efficient gelatinase inhibitor when compared with inorganic fluorides (Mei et al., 2012).

It can be hypothesized that the inhibition of NCP cleavage by MMP-3 inhibitors may prevent further matrix degradation by protecting the collagen fibers from collagen-specific MMPs such as collagenases and gelatinases. Indeed, PGs were initially reported as the major substrates of MMP-3. However, the situation may be more complex since PGs are bound to several other proteins in the extracellular matrix (Qin et al., 2006). For example, MMP-9 is linked to the core protein of Chondroitin sulfate proteoglycans, forming a pro-MMP-9-PG heteromer (Winberg et al., 2003; Malla et al., 2008). Although there is no literature reporting such complex in the dentin extracellular matrix, it cannot be excluded that the cleavage of this hetereomer by MMP-3 may release active MMP-9 (Malla et al., 2008) which could in turn express its gelatinolytic activity, exacerbating the dentin matrix degradation initiated by MMP-3.

In conclusion, the present study shows that the combination of GSE and AmF as in the evaluated mouthrinse limits dentin matrix degradation. This association may be promising in the prevention of caries. However, the procedure should be adapted to durations that are clinically relevant.

### Conflict of interest statement

The authors declare that the research was conducted in the absence of any commercial or financial relationships that could be construed as a potential conflict of interest.
